# Unravelling the Persistence of the Rare Serovar *Salmonella* Mikawasima in a Hospital Setting: A Whole-Genome Sequencing Study

**DOI:** 10.3390/pathogens14050408

**Published:** 2025-04-24

**Authors:** Ivana Ferencak, Ana Gveric Grginic, Tajana Juzbasic, Irena Tabain, Marija Tonkic, Ivana Goic-Barisic, Dragan Juric, Hrvojka Jankovic, Luka Katic, Anita Novak

**Affiliations:** 1 Department of Microbiology, Croatian Institute of Public Health, 10000 Zagreb, Croatia; ana.gveric-grginic@hzjz.hr (A.G.G.); tajana.juzbasic@hzjz.hr (T.J.); irena.tabain@hzjz.hr (I.T.); dragan.juric@hzjz.hr (D.J.); hrvojka.jankovic@hzjz.hr (H.J.); 2 Food-and Water-Borne Infections Study Group (EFWISG), European Society of Clinical Microbiology and Infectious Disease (ESCMID), 4051 Basel, Switzerland; luka.katic@mountsinai.org (L.K.); annovak@kbsplit.hr (A.N.); 3 Department of Clinical Microbiology, University Hospital of Split, 21000 Split, Croatia; mtonkic@kbsplit.hr (M.T.); igoic@kbsplit.hr (I.G.-B.); 4 School of Medicine, University of Split, 21000 Split, Croatia; 5 Department of Medicine, Icahn School of Medicine at Mount Sinai Morningside/West, 1000 Tenth Avenue, New York, NY 10019, USA

**Keywords:** *S*. Mikawasima, endemics, virulence, biofilm, antimicrobial resistance, whole-genome sequencing, healthcare-associated infections

## Abstract

*Salmonella* Mikawasima is a rare, mainly environmental serovar. In 2020, an outbreak was observed in neonatal and maternal wards of the University Hospital of Split and was established as an endemic until the end of 2024. Using whole-genome sequencing, this study aimed to analyse the phenotypic and genotypic characteristics of *S*. Mikawasima isolates and to elaborate whether the spread of the same clone occurred. Sequenced isolates were classified as ST2030, with the presence of aminoglycoside and extended spectrum beta-lactam resistance genes. Ten percent of the sequenced isolates exhibit multi-drug resistance. Identified virulence factors that include biofilm formation genes suggest the potential persistence of *S*. Mikawasima in the hospital environment, while spatial and temporal analysis reveal clonal expansion and possible horizontal transmission between different hospital wards. This study provides a deep understanding of the genomic composition of *S*. Mikawasima and emphasises the need for more stringent infection prevention measures, especially in vulnerable neonatal and postpartum settings, to mitigate the risk of healthcare-associated infections, and it should be followed by further microbiological and epidemiological investigations to identify the source of infection.

## 1. Introduction

*Salmonella* (*S.*) Mikawasima is a rare serovar belonging to *S. enterica* subspecies *enterica*. It is a part of the O:7 (C1) serogroup, showing antigenic profile formula O 6,7,14: y: e, n, z15 [1,2]. Its occurrence was firstly described in Turkey in 1967, when a previously unknown serovar was found in the digestive system of turtles. Since then, it has been found in Turkey in frog liver and in wild boars and birds in the north and south of Spain [3,4,5,6]. Apart from scarce reports of identification in animals and environmental and food samples, *S.* Mikawasima is also a rare cause of human salmonellosis outbreaks.

According to the European Food Safety Authority (EFSA) and European Centre for Disease Prevention and Control (ECDC) technical report and rapid outbreak assessment published in 2013, 671 human cases of *S*. Mikawasima were documented in EU/EEA countries and submitted to the European Surveillance System (TESSy) between 2007 and 2012 [7]. In the autumn of 2013, there was a major outbreak in the United Kingdom (U.K.), with 75 reported cases. It was likely a multistate outbreak with an unknown source. The case distribution exhibited significant seasonality across months, characterized by consistent peaks from September to November. It occurred mostly in adults, with at least 65% of cases documented in individuals over 25 years of age; there was no substantial variation between genders throughout the years, and there was a rather stable male-to-female ratio [7].

The next big cross-border outbreak was noted in 2019. The outbreak was first reported in the UK with 138 cases, Sweden with 31 cases, France with 18, Denmark with 2, and Ireland with 1 case. The epidemiologic data lack a complete travel history, and investigations pointed to the possible vehicle of infection distributed in different EU countries [8]. At the end of 2024, there was again a cross-border outbreak in Europe. Recent epidemiologic data from TESSy suggest travel-related infections, and also an infant community, but the details are not yet publicly available [9].

Reports on *Salmonella* species (spp.) as a causative agent of infections that are acquired during medical treatment in various healthcare facilities (healthcare-associated infections, HCAIs) are scarce [10,11,12,13].

The endemic occurrence of the *S*. Mikawasima in departments related to maternity and neonatal care at the University Hospital of Split (UHS) in the southern coast region of Croatia since April 2020 raises concerns [14], and it is necessary to employ all methods available to modern microbiological laboratories to gain high-resolution insights into the bacterial defence and virulence mechanisms so that, in synergy with clinical data, we can act on the source of infection and interrupt transmission pathways.

The aims of our study were to determine the phenotypic and genotypic characteristics of isolated strains of *S*. Mikawasima in UHS, to identify virulence and antimicrobial resistance (AMR) genes, to establish the relationship between the isolated strains, and to verify whether the isolates are part of the ongoing endemics or whether there were new entries and reinfections with different strains from the community. The specific research objectives included the application of whole-genome sequencing (WGS) and mutual comparison of genomes through core genome multi locus sequence typing (cgMLST). Because of the importance of understanding and dealing with the continued persistence of this rare serovar in neonatal hospital settings, this paper is a continuation of the study carried out by Novak at al., which showed that WGS can be a good tool for managing hospital infections [14].

We envisioned that managing this epidemic could benefit from our HERA2 project, which has, as one of its outcomes, scaling-up WGS capacities in outbreak detection, and that providing evidence of persistence can help in improving infection control measures and thus bring this epidemic to a close.

## 2. Materials and Methods

### 2.1. Study Design

This retrospective observational study was conducted at the Department of Clinical Microbiology, UHS, Croatia, from August 2023 to October 2024, and in parallel at the National Reference Laboratory for Salmonellae (NRLSalm), Division of Microbiology, Croatian Institute of Public Health (CIPH), Croatia.

### 2.2. Clinical Cases and Isolates

Bacterial strains were obtained from stool samples, rectal and throat swabs, and haemoculture collected during the study period from neonatal, paediatric, and maternal wards at UHS. The samples were then processed in the microbiological laboratory. Strains identified as *S*. Mikawasima were sent to NRLSalm for further genomic characterization. Only successfully sequenced strains that satisfied quality control criteria were included in this study.

### 2.3. Biochemical Characterization and Antimicrobial Susceptibility Testing

Initial samples were cultivated on Salmonella Shigella agar plates (Liofilchem, Roseto degli Abruzzi, Italy), and presumptive colonies were identified to species level using MALDI-TOF mass spectrometry (microflex^®^ LRF; Bruker, Berlin, Germany). Strains identified as *S. enterica*, subsp. *enterica*, were serotyped according to White–Kaufmann–Le minor Scheme [1,2], using the slide agglutination technique with specific antisera (Sifin diagnostics gmbh, Berlin, Germany) for somatic O and flagellar H antigens. Antimicrobial susceptibility testing was conducted using the disk-diffusion method and Vitek 2 Compact system (bioMérieux, Lyon, France) with an AST-N379 card. Testing and interpretation were performed using European Committee on Antimicrobial Susceptibility Testing (EUCAST) breakpoints (v.13.1, and 14.0) [15]. The antimicrobial agents tested included ampicillin, ceftriaxone, ceftazidime, amoxicillin + clavulanic acid, chloramphenicol, trimethoprim-sulfamethoxazole, and pefloxacin as screening for ciprofloxacin. Extended-spectrum beta lactamase (ESBL) production in isolated strains was performed by double disk-diffusion test, using cefotaxime (CTX) and ceftazidime (CAZ) disks, with and without clavulanic acid (CLV), and *Escherichia coli* ATCC 25922 for the control.

### 2.4. Whole-Genome Sequencing and Bioinformatic Analysis

Upon identification, 38 isolated strains were successively referred to NRLSalm for further characterisation. DNA was extracted from pure bacterial cultures using the NucleoSpin Tissue kit (Macherey-Nagel, Dueren, Germany) following the manufacturer’s protocol, which included a 3 h proteinase K digestion at 56 °C. A WGS library with 2 × 150 bp reads was prepared using the Illumina DNA Prep kit and sequenced on an Illumina NextSeq 550 instrument (Illumina, San Diego, CA, USA). After sequencing, quality control was performed on acquired reads using FastQC v0.12.1, followed by read trimming with Fastp v0.23.4 [16,17]. De novo genome assembly was performed using the SPAdes genome assembler v3.10.1 with default parameters and the “careful” option [18]. The assembled genomes were then evaluated for quality and G+C content using Quast software v5.2.0 and Samtools v1.21 [19,20]. The analytical procedure adhered to acceptable quality control (QC) standards throughout the process. The samples that were not in line with quality parameter acceptance criteria (less than 4% deviation from the expected G+C content for species analysed, and total length of all contigs approximated to the known genome size of the target organism) were excluded from the study. All metrics related to the quality control and assembly are included as Supplementary Table S1.

The data generated in this study have been submitted to the European Nucleotide Archive (ENA).

Assembled genomes that passed quality requirements were identified against the RefSeq database with RefSeq masher v0.1.2 [21,22]. The reference genome used for *Salmonella* spp. analysis in our laboratory is GCF_000027025.1 [23]. Detailed serotype prediction was achieved using sistr_cmd v1.1.3 [24]. The multi-locus sequence types (MLSTs) were identified using mlst v2.23.0 [25]. High-resolution bacterial strain typing and evolutionary analysis by comparing genetic variations of conserved gene loci across genome (cgMLST), was performed using Ridom SeqSphere+ v4.0 [26]. This analysis was performed on our sequences but also on sequences obtained from the beginning of the epidemic and a set of publicly available *S*. Mikawasima sequences [14]. The distance matrices were visualised using the GrapeTree and iTOL online tools [27,28].

AMR mechanisms were determined using ResFinder v4.7.2 software that analyses de novo genome assemblies against ResFinder database: (2024-03-22) and PointFinder database: (2024-03-08) [29,30]. The analysis was conducted using a 90% identity threshold and 60% minimum length. Plasmids were searched using PlasmidFinder v2.1 with database version v2023-01-18 [31]. Mapping of resistance genes against plasmid sequences was performed using Mob-suite v3.1.9 and MobileElementfinder v1.0.3 with database v1.0.2 [32,33]. Abricate v1.0.1 was employed against VFDB database v20253-02-05 for identification of genes associated with virulence, and SPIFinder v2.0 was used to identify *Salmonella* spp. pathogenicity islands by running sequences against Pathogenic islands and resistance islands database (PAIDB) v2.0 [34,35,36,37].

## 3. Results

### 3.1. Isolates Metadata

Among 4979 tested stool samples and rectal swabs in UHC during the study period, 36 were positive for *S.* Mikawasima. Other serovars were sporadically isolated throughout the UHC, but none at the neonatal and maternal units, and they were not part of this study. One *S.* Mikawasima strain was isolated from a haemoculture, and one from a throat swab. Available information related to individual strains containing strain code, sampling dates, patient age, associated symptoms, hospital units, and antimicrobial resistance results are presented in Table 1.

### 3.2. Genomic Relatedness

**Figure 1:**
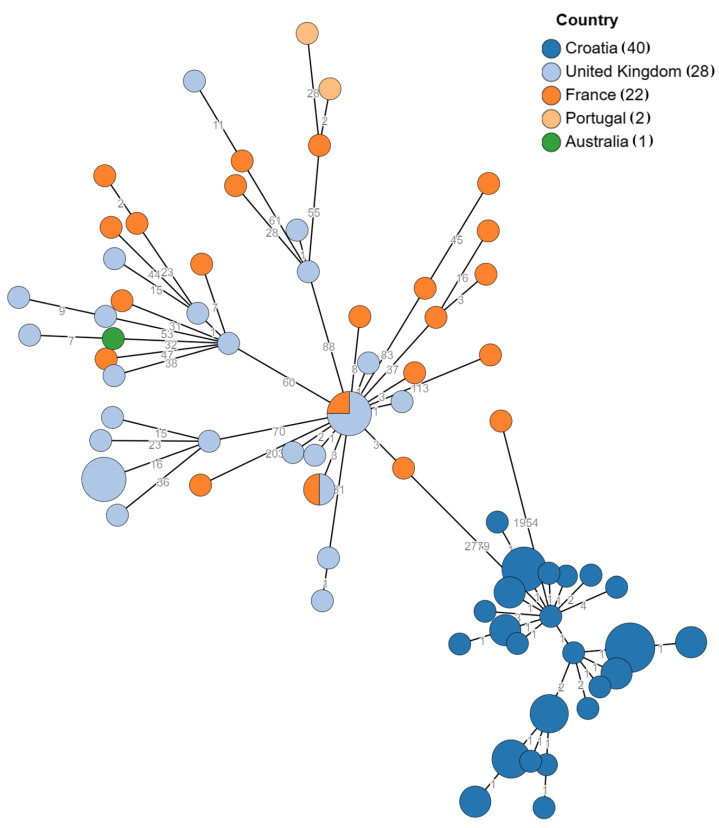
GrapeTree representation of cgMLST allelic distances between our strains and publicly available sequences. Branch lengths do not reflect the degree of sequence divergence because of the log scale representation chosen to show genetically similar sequences. Labels on branches represent number of different loci, and the size of the node represents the number of sequences without allelic differences.

All isolates were identified as *S. enterica*, subsp. *enterica*, and serotype was predicted as *S.* Mikawasima, as were identified in UHS using the slide agglutination technique, showing a 7:y:e,n,z15 antigenic profile. The MLST scheme places all isolates in sequence type (ST) 2030. The distance matrix created with publicly available *S.* Mikawasima sequences from Enterobase and three sequences from the Novak at al. study shows that all isolates are a part of the same epidemiologic cluster, which is shown in Figure 1 [14,26].

**Figure 2:**
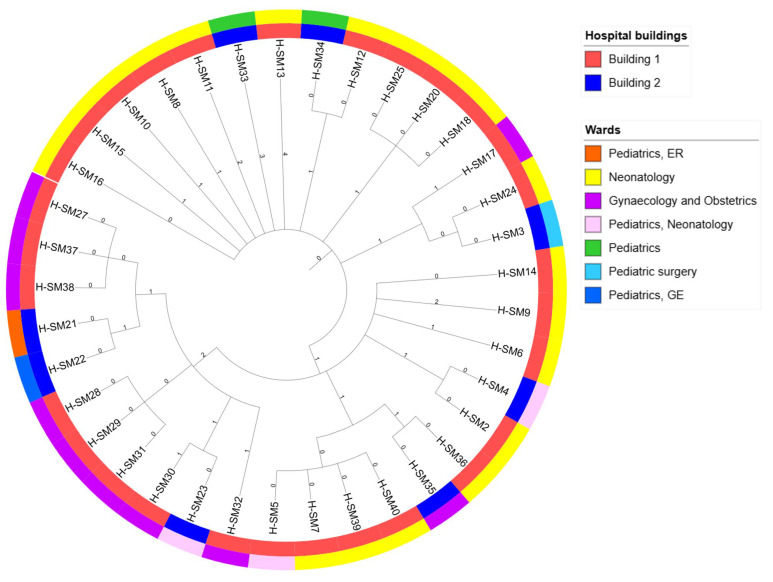
Circular phylogenetic tree illustrating the genetic relationships among isolates. Each node corresponds to a specific isolate. Branch lengths do not reflect the degree of sequence divergence. Labels on branches represent number of different loci, and the legend depicts different hospital wards.

To visualise relatedness of different *S*. Mikawasima strains, and to demonstrate their intrahospital location, we used the iTOL visualisation tool [28]. The phylogenetic tree is shown in Figure 2.

The distribution of samples, phenotypic ESBL patterns, and timelines according to occurrence in different hospital buildings and hospital units are shown in Table 2. It is visible from the table that majority of samples were isolated in the Neonatology Unit and closely related Gynaecology and Obstetrics Unit, placed in the same hospital building (Building 1). Sporadic but genetically closely related isolates were isolated from other buildings (Building 2) but from wards with a connection to the Neonatology Unit.

### 3.3. AMR Gene Profile and Phenotypic Correlation

All isolates harbour genes for aminoglycoside resistance, namely N-Acetyltransferases (aac): *aac(6′)-Iaa*, *aac(6′)-Im and aph(2″)-Ib*, (37/37, 100%).

Genes encoding beta-lactamase enzymes *bla*_TEM-1B_ and *bla*_SHV-2_, and *bla*_TEM-1B_ and *bla*_SHV-2_ together, are detected in 14/37 (37.8%), 6/37 (16.2%), and 17/37 (45.9%), respectively. Phenotypic expression of extended spectrum beta-lactamase was identified in 16 (43.3%) isolates.

Four out of thirty-seven (10.8%) isolates have the *tet(D)* gene that confers tetracycline resistance. These four isolates (H_SM3, H_SM22, H_SM33, and H_SM_34) are thus genotypically classified as multiple drug resistance (MDR) strains. All isolates carry the incompatibility group C plasmid (IncC) that can carry multiple resistance genes. These genes are often located within transposon integrons and other mobile genetic elements [38]. Mapping of resistance genes against the IncC plasmid sequence revealed that *aac(6′)-Im* and *aph(2″)-Ib, bla_TEM-1B_* are present on the IncC plasmid, while *aac(6′)-Iaa* is chromosomally encoded. The same is true for *bla*_TEM-1B_ and *tet(D)* genes. In Table 3, the phenotypic antimicrobial susceptibility pattern and its correlation with the results of beta-lactamase profiling are shown. According to EUCAST guidelines, and based on clinical relevance, *Salmonella* spp. isolates are not phenotypically tested for susceptibility to aminoglycoside and tetracycline [15].

### 3.4. Virulence Genes as Biomarkers of Persistence

All isolates carry the following four *Salmonella* pathogenicity islands (SPI): SPI-1, SPI-2, SPI-3, and SPI-13.

Detected virulence genes are grouped in Table 4 into categories according to the VFDB database [39]. All the stated virulence factors are detected in all the tested strains. A complete list of detected virulence genes is presented in Supplementary Table S2.

## 4. Discussion

Data presented in this study show the complex behaviour of rarely detected *Salmonella* Mikawasima in a hospital environment. Distribution of samples between specific hospital units, the timeline of the isolates’ detection, and their correlation with the sequences deposited in GenBank from the Novak et al. study [14] suggest clonal expansion of ST 2030 in the same hospital settings from April 2020 until the end of 2024. Our research is a continuation of an earlier study due to the need for a deeper insight into the genomic characteristics of novel isolates throughout 2024 and in response to the dilemma whether this corresponds to the possible introduction of new clones from the community or further spread of the same clone within the hospital’s neonatology and paediatric settings.

The repeated detection of ESBL-positive ST 2030 in the same units over the studied period shows that *S*. Mikawasima became endemic in these specific units. Within all other hospital wards and units there were no *S*. Mikawasima isolates. Additionally, there were no other *Salmonella* spp. serovars isolated in the neonatal and paediatric settings in UHS.

Published reports on epidemic occurrences of *Salmonella* spp. in neonatal and paediatric hospital settings are scarce and include other different causative serovars. *S*. Tennessee caused epidemics in a neonatal intensive care unit in the U.S.A. and was connected to national epidemics linked to contaminated peanut butter. Multidrug-resistant *S*. Typhimurium caused prolonged two-year nosocomial epidemics in a paediatric ward in South Africa, and some authors hypothesised that the causative agent was introduced into the hospital setting from the community. The U.K.’s *S*. Enteritidis epidemic that affected mothers and new-borns was most likely initially spread by contaminated resuscitator equipment [11,12,13].

Detection of the same *S.* Mikawasima sequence type in other hospital units that include paediatric and gynaecology departments, even in two different buildings, suggests its ability to spread rapidly between different settings in a hospital environment. These other affected units handle postnatal care of neonates and mothers, indicating horizontal transfer between these wards, possibly through patient transfers, shared HCWs, or contaminated equipment.

Besides ST 2030 circulating in Europe, ST 1815 is an even more prominent *S*. Mikawasima sequence type that caused a cross-border outbreak in November 2019 [8]. At the end of 2024, there was again a cross-border outbreak of ST 1815 in Europe [9].

*S*. Mikawasima, as a relatively rare serovar, was not of particular interest in molecular studies of S*almonella* spp. Apart from the Novak at al. paper [14], *S*. Mikawasima was described in the Joint EFSA and ECDC Rapid Outbreak Assessment after the 2013 increase in human infections in Europe [7]. They focused mainly on Pulse Field Gel Electrophoresis (PFGE) findings, as WGS was not widely available, and PFGE was still the gold standard in typing of *Salmonella* spp. [41]. An outbreak in Denmark, as a part of this 2013 increase, was analysed by WGS, but only for cluster detection [42]. Our study fulfils this knowledge gap and provides a deeper understanding of *S*. Mikawasima genomic composition and behaviour in a hospital environment.

The persistence of *S*. Mikawasima in hospital environments could be attributed to its formation and survival in biofilm outside of a human host. Our analysis revealed several adherence factors that are important for creation and survival of *S*. Mikawasima in a hospital environment. Curli fimbriae genes (*csgA*, *csgB*, *csgC*, *csgE*, *csgF*, *csgG*) are controlled by *csgD* and are critical in attachment to surfaces like stainless steel and plastics because of their role in the production of curli fimbriae and cellulose [43,44]. These fimbriae are more expressed when bacteria resides in a nutrient-depleted environment [45,46]. *FimH* and other type 1 fimbriae genes are important for adherence to different surfaces [47]. The *Mig-14* gene, besides participating in polymyxin B resistance, promotes biofilm formation in harsh environments by regulating the expression of different virulence factors [40]. Although in this study biofilm formation assay was not performed, detection of these virulence genes indicates the genetic potential for environmental survival due to contamination and the potential for a permanent reservoir of *S*. Mikawasima in the hospital environment and/or medical equipment.

The presence of SPI-1 and SPI-2 points towards a type III secretion system (T3SS) that contributes to rapid intestinal colonisation, inflammation, and increased transmission in hospital environments [48,49]. *Salmonella* strains that do not carry SPI-1 and SPI-2 cannot induce inflammation [50]. There are studies that suggest that strains that carry SPI-1 display biofilm formation in media [51]. The function of SPI-3 is not so clear, but the data suggest that deletion of any of these three SPIs results in a reduction in adhesion capacity and possibly in biofilm formation [52]. SPI-13 contributes to intracellular survival of the bacteria [53].

The antimicrobial phenotypic beta-lactam resistance patterns show constant resistance to ampicillin and amoxicillin-clavulanic acid across almost all isolates (37/37, 100%). Exhibited phenotypic resistance levels to amoxicillin-clavulanate are slightly outside the 100% range (35/37, 94,6%). These phenotypic patterns of resistance are backed up by the detection of *bla*_SHV-2_ and *bla*_TEM-1_ genes. While in our strains, *bla*_SHV-2_ is chromosomally encoded, and *bla*_TEM-1_ is transferred across species by a broad host range of IncC plasmids that all our isolates carry.

In addition to resistance to aminopenicillins with or without beta-lactamase inhibitors, ESBL-positive strains express resistance to third generation cephalosporins with single strain resistance to ciprofloxacin (1/37, 0.93%). This specific case of phenotypic resistance does not have an underlying detected genetic basis of gene mutation confirmed through whole-genome sequencing. This warrants further investigation.

The presence of the gene *tet(D)* that confers resistance to tetracycline in four isolates, together with aminoglycoside resistance genes, designates them genotypically as MDR microorganisms, which diminishes treatment options, especially in vulnerable neonate patients for whom ampicillin in combination with aminoglycosides is a first-line empiric treatment [54]. The *tet(D)* gene is the least studied *tet* gene, encoding an efflux pump that expels tetracycline from bacterial cells in *Salmonella* isolates [55]. Its presence in four among a cluster of closely related strains remains a topic for further analysis, especially considering its chromosomal position. Different mobile genetic elements (insertion sequences, inverted repeats) detected throughout the genome might, in settings like the one described in this study, with the indication of continuous contamination and selective antimicrobial pressure, pose a high risk for persistence and spread of MDR strains, especially due to the presence of IncC plasmid. As cgMLST analysis considers only conserved regions of the genome, strains with no cgMLST allelic difference could have a different AMR profile.

Hospitals are a fundamental part of healthcare system, presenting a setting in which the most diversified and advanced treatment at the highest level is performed. This poses a risk for bacterial transmission and the emergence of healthcare-associated infections. Individuals at age extremes, including neonates and the elderly, are most susceptible to acquiring HCAIs. Prevalence rates of HCAIs and their bacterial causative agents in neonates vary across different hospital settings. As determined by ECDC point-prevalence survey of healthcare-associated infections in acute care hospitals conducted during 2011 and 2012 in 1149 hospitals across EU Member States, Iceland, Norway, and Croatia, prevalence of HCAIs in neonatology units was 3.5% (CI 95% 2.8–4.5%), paediatric surgery units 3.4% (CI 95% 2.3–4.9%), and general paediatric units 1.8% (CI 95% 1.4–2.4%) The prevalence rate of gastrointestinal infections acquired during hospitalization was 8.3% (CI 95% 6.4–10%). Although *Enterobacterales* were the most isolated microorganisms (15%) among detected pathogens causing all HCAIs, no *Salmonella* species was reported [56,57]. Another ECDC point-prevalence survey was conducted in the EU/EEA, including Croatia, during 2016/2017 [58], and according to the unpublished data from that study, the prevalence of HCAIs in Croatian paediatric and neonatal units was 5.2% and 1.8%, respectively. Among bacterial causative agents, no *Salmonella* species was detected.

Preventing the emergence of HCAIs, their epidemic or endemic occurrence, as well as interrupting the spread of already established ones, is only possible with a thorough understanding of the mechanisms of virulence and the antibiotic resistance that bacterial pathogens develop and utilize to successfully evade the effects of therapy and disinfection, thus maintaining themselves in the population and/or environment.

Our study indicates that, although *S.* Mikawasima is a rare serovar usually present in animals and contaminated environmental and food samples, it can emerge as a significant cause of hospital-acquired infections, especially in sensitive populations. It also can cause prolonged endemics in hospital settings. In Split-Dalmatia (SD) county, unlike other parts of Croatia, *S*. Mikawasima is the most frequently isolated serovar of *Salmonella* spp. [59,60]. Further genomic studies are needed to establish routes of transmission, the source of the infection, and to stop the spreading in hospital settings, as well as in the community.

The main drawback of this study is the lack of environmental samples. These samples were taken and processed in the local public health institute, and according to unofficial information, no source has been revealed. It is our hope that based on our research, which suggests the clonal spread within different and remote hospital settings, a more detailed investigation will be conducted, bearing in mind the behaviour of *S*. Mikawasima. It is evident that more effort is needed to implement rigorous measures for preventing HCAIs, particularly in neonatal care units where continuous antibiotic and disinfectant usage selects bacteria with a competitive advantage for survival in the hospital environment achieved by acquiring genes and mutations of antimicrobial resistance and different virulence factors, which we were able to show through means of analysis of WGS data.

Additionally, infection control and prevention measures should be conducted not only within a particular unit’s level but also at the hospital level, with the inclusion of all patient—HCW—equipment pathways. Comprehensive research across all sectors using the “One Health” approach is recommended to better understand and evaluate the risks linked to *S*. Mikawasima spreading.

## Figures and Tables

**Table 1:** Detailed metadata related to individual strains.

Strain Code	Sampling Date	Age/Months	Sample	Symptoms	Unit	AMR Profile *	ESBL
H_SM2	August 2023	<1	stool	diarrhoea	Neonatology	AM, AMC, CRO, CAZ, CTX	positive
H_SM3	October 2023	13	stool	diarrhoea	Clinic for paediatric surgery, Department for abdominal surgery	AM, AMC	negative
H_SM4	November 2023	<1	stool	diarrhoea	Paediatrics, neonatology	AM, AMC, CTX, CAZ, CIP	positive
H_SM5	November 2023	1	stool	diarrhoea	Paediatrics, neonatology	AM, AMC, CRO, CAZ, CTX	positive
H_SM6	November 2023	<1	stool	diarrhoea	Neonatology	AM, AMC, CRO, CAZ, CTX	positive
H_SM7	November 2023	<1	stool	diarrhoea	Neonatology	AM, AMC, CTX, CAZ	positive
H_SM8	January 2024	<1	rectal swab	diarrhoea	Neonatology	AM, AMC, CTX	positive
H_SM9	January 2024	<1	rectal swab	diarrhoea	Neonatology	Missing data	positive
H_SM10	January 2024	<1	rectal swab	diarrhoea	Neonatology	AM, AMC, CTX	positive
H_SM11	January 2024	2	rectal swab	diarrhoea	Neonatology	AM, AMC, CTX	positive
H_SM12	January 2024	1	throat swab	asymptomatic colonisation	Neonatology	AM, AMC	positive
H_SM13	January 2024	<1	rectal swab	diarrhoea	Neonatology	AM, AMC, CTX	positive
H_SM14	January 2024	<1	rectal swab	diarrhoea	Neonatology	AM, AMC	negative
H_SM15	January 2024	1	rectal swab	diarrhoea	Neonatology	AM, AMC, CAZ	positive
H_SM16	February 2024	3	rectal swab	diarrhoea	Neonatology	AM	positive
H_SM17	February 2024	<1	stool	diarrhoea	Gynaecology and Obstetrics	AM, AMC, CTX	positive
H_SM18	December 2023	2	rectal swab	diarrhoea	Neonatology	AM, AMC	positive
H_SM20	February 2024	<1	rectal swab	diarrhoea	Neonatology	AM, AMC, CRO	positive
H_SM21	March 2024	1	stool	diarrhoea	Paediatrics, ER	AM, AMC	negative
H_SM22	March 2024	1	stool	diarrhoea	Paediatrics, GE	AM, AMC	negative
H_SM23	March 2024	3	stool	diarrhoea	Paediatrics, neonatology	AM, AMC	positive
H_SM24	July 2024	<1	haemoculture	fever	Neonatology	AM, CAZ	positive
H_SM25	July 2024	<1	stool	diarrhoea	Neonatology	AM, CTX, CAZ	positive
H_SM27	July 2024	<1	rectal swab	diarrhoea	Gynaecology and Obstetrics	AM, AMC	negative
H_SM28	August 2024	<1	stool	diarrhoea	Gynaecology and Obstetrics	AM, AMC	negative
H_SM29	August 2024	<1	stool	diarrhoea	Gynaecology and Obstetrics	AM, AMC	negative
H_SM30	August 2024	<1	rectal swab	diarrhoea	Gynaecology and Obstetrics	AM, AMC	negative
H_SM31	August 2024	<1	stool	diarrhoea	Gynaecology and Obstetrics	AM, AMC	negative
H_SM32	August 2024	<1	stool	diarrhoea	Gynaecology and Obstetrics	AM, AMC	negative
H_SM33	July 2024	1	stool	diarrhoea	Paediatrics	AM, CTX	positive
H_SM34	July 2024	<1	stool	diarrhoea	Paediatrics	AM, CAZ	positive
H_SM35	September 2024	<1	rectal swab	diarrhoea	Gynaecology and Obstetrics	AM, AMC	negative
H_SM36	September 2024	3	rectal swab	diarrhoea	Neonatology	AM, AMC, CTX, CAZ	positive
H_SM37	September 2024	<1	rectal swab	diarrhoea	Gynaecology and Obstetrics	AM, AMC	negative
H_SM38	September 2024	<1	stool	diarrhoea	Gynaecology and Obstetrics	AM, AMC	negative
H_SM39	September 2024	<1	rectal swab	diarrhoea	Neonatology	AM, AMC, CTX, CAZ	positive
H_SM40	October 2024	<1	stool	diarrhoea	Neonatology	AM, AMC	negative

* AM—amikacin, AMC—amoxicillin + clavulanic acid, CAZ—ceftazidime, CTX—Cefotaxime, CRO—ceftriaxone.

**Table 2:** Timeline and distribution of isolates according to occurrence in two different buildings in the hospital setting.

	August 2023	October 2023	November 2023	December 2023	January 2024	February 2024	March 2024	July 2024	August 2024	September 2024	October 2024
**Building 2**											
Paediatric, neonatology unit			2				1				
Paediatric, GE unit							1				
Paediatric, ER							1				
Clinic for paediatric surgery, Department for abdominal surgery		1									
Paediatric								2			
**Building 1**											
Neonatology Unit	1		2	1	7 1	2		2		2	1
Gynaecology and Obstetrics Unit						1		1	5	3	

▪ ESBL-positive strains; ▪ ESBL-negative strains.

**Table 3:** Correlation of AMR phenotype, ESBL production, and detected beta-lactamase genes.

ESBL	AMR Phenotypic Pattern	*bla*_TEM-1B_	*bla*_SHV-2_	*bla*_TEM-1B_ and *bla*_SHV-2_	No. of Isolates
ESBL-negative	Ampicillin, amoxicillin-clavulanate	13	1		14
ESBL-positive	Ampicillin			1	1
	Ampicillin, amoxicillin-clavulanate	1		2	3
	Ampicillin, amoxicillin-clavulanate, cefotaxime			5	5
	Ampicillin, amoxicillin-clavulanate, cefotaxime, ceftazidime		1	2	3
	Ampicillin, amoxicillin-clavulanate, cefotaxime, ceftazidime, ciprofloxacin			1	1
	Ampicillin, amoxicillin-clavulanate, ceftazidime			1	1
	Ampicillin, amoxicillin-clavulanate, ceftriaxone			1	1
	Ampicillin, amoxicillin-clavulanate, ceftazidime, cefotaxime, ceftriaxone			3	3
	Ampicillin, cefotaxime		1		1
	Ampicillin, cefotaxime, ceftazidime		1		1
	Ampicillin, ceftazidime		2		2
Missing phenotypic data				1	1

**Table 4:** Categories of detected virulence genes.

Virulence Genes	
Adherence	*csgA*, *csgB*, *csgC*, *csgD*, *csgE*, *csgF*, *csgG*, *fimC*, *fimD*, *fimF*, *fimH*, *fimI*, *misL*, *sinH*, *steA*, *steB*, *steC*,
Effector delivery system	*sipA*, *sipB*, *sipC*, *sipD*, *invC*, *sopA*, *sopB*, *sopD*, *sopD2*, *sopE2*, *prgH*, *prgI*, *prgJ*, *prgK*, *invA*, *invB*, *invE*, *invF*, *invG*, *invH*, *invI*, *invJ*, *sicA*, *sicP*, *spaO*, *spaP*, *spaQ*, *spaR*, *spaS*, *ssaA*, *ssaB*, *ssaC*, *ssaD*, *ssaE*, *ssaF*, *ssaG*, *ssaH*, *ssaI*, *ssaJ*, *ssaK*, *ssaL*, *ssaM*, *ssaN*, *ssaO*, *ssaP*, *ssaQ*, *ssaR*, *ssaS*, *ssaT*, *ssaU*, *ssaV*, *sscA*, *sscB*, *orgA*, *orgB*, *orgC*, *sptP*, *sseJ*, *sseK2*, *sseL*, *pipB*, *pipB2*, *sifA*, *sifB*, *avrA*, *spiC/ssaB*, *sprP*
Exotoxin	*cdtB*
Nutritional/Metabolic factor	*mgtC*, *mgtB*
Antimicrobial activity and biofilm formation [40]	*mig-14*

## Data Availability

The original data presented in the study are openly available from the European Nucleotide Archive at https://www.ebi.ac.uk/ena/browser/view/PRJEB86855 (accessed on 28 March 2025).

## Supplementary Materials

The following supporting information can be downloaded at: https://www.mdpi.com/article/10.3390/pathogens14050408/s1.
